# Structure of the imine reductase from *Ajellomyces dermatitidis* in three crystal forms

**DOI:** 10.1107/S2053230X23006672

**Published:** 2023-08-15

**Authors:** Mahima Sharma, Anibal Cuetos, Adam Willliams, Daniel González-Martínez, Gideon Grogan

**Affiliations:** a Department of Chemistry, University of York, Heslington, York YO10 5DD, United Kingdom; University of Leipzig, Germany

**Keywords:** imine reductases, biocatalysis, *Ajellomyces dermatitidis*

## Abstract

The NADPH-dependent imine reductase from *Ajellomyces dermatitidis* (*Ad*RedAm) catalyzes the reductive amination of certain ketones with amine donors supplied in an equimolar ratio. The structure of *Ad*RedAm has been determined in three forms. The first form, which belongs to space group *P*3_1_21 and was refined to 2.01 Å resolution, features two molecules (one dimer) in the asymmetric unit in complex with the redox-inactive cofactor NADPH_4_. The second form, which belongs to space group *C*2_1_ and was refined to 1.73 Å resolution, has nine molecules (four and a half dimers) in the asymmetric unit, each complexed with NADP^+^. The third form, which belongs to space group *P*3_1_21 and was refined to 1.52 Å resolution, has one molecule (one half-dimer) in the asymmetric unit. This structure was again complexed with NADP^+^ and also with the substrate 2,2-difluoroacetophenone. The different data sets permit the analysis of *Ad*RedAm in different conformational states and also reveal the molecular basis of stereoselectivity in the transformation of fluorinated acetophenone substrates by the enzyme.

## Abstract 2

The structure of the imine reductase from *A. dermatitidis* is presented in three crystal forms, each of which provides information on conformational dynamics and cofactor and substrate binding within the enzyme.

## 1. Introduction

**Figure 1:**
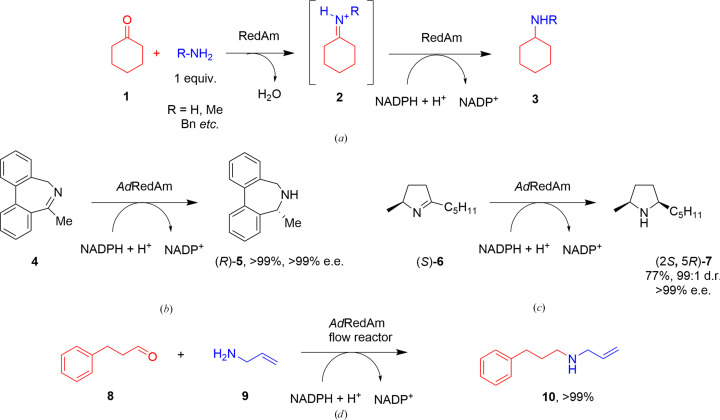
(*a*) Reductive amination reactions catalyzed by RedAms. (*b*) Reduction of dibenzazepine (**4**) by *Ad*RedAm (France *et al.*, 2017 ▸). (*c*) Reduction of pyrrolidine (**6**) by *Ad*RedAm (Costa *et al.*, 2018 ▸). (*d*) Reductive amination of hydrocinnamaldehyde (**8**) with allylamine (**9**) catalyzed by *Ad*RedAm in a flow reactor (Finnigan *et al.*, 2020 ▸).

Chiral amines are important functionalities in biologically active molecules and their asymmetric synthesis by reductive amination is a significant reaction in industrial medicinal chemistry (Afanasyev *et al.*, 2019 ▸). Recently, a subset of NADPH-dependent imine reductase (IRED; Grogan & Turner, 2016 ▸; Mangas-Sanchez *et al.*, 2017 ▸) enzymes have been identified that catalyze the reductive amination of a range of ketones with small amine donors when supplied at one stoichiometric equivalent (Fig. 1 ▸ *a*; Aleku *et al.*, 2017 ▸; Gilio *et al.*, 2022 ▸). These ‘reductive aminases’ (RedAms; Aleku *et al.*, 2017 ▸; Gilio *et al.*, 2022 ▸) have now been applied in the multi-kilogram (Schober *et al.*, 2019 ▸) and ton (Kumar *et al.*, 2021 ▸) scale synthesis of pharmaceutical precursors by many industrial (Schober *et al.*, 2019 ▸; Kumar *et al.*, 2021 ▸; Ma *et al.*, 2021 ▸) and academic (Mangas-Sanchez *et al.*, 2020 ▸; Yang *et al.*, 2021 ▸; Zhang *et al.*, 2022 ▸; Chen *et al.*, 2023 ▸; Gilio *et al.*, 2023 ▸) groups. RedAms were first identified in enzymes from fungi, and structures of two of them, *Asp*RedAm from *Aspergillus oryzae* (Aleku *et al.*, 2017 ▸) and *At*RedAm from *A. terreus* (Sharma *et al.*, 2018 ▸), each in complex with the NADP^+^ cofactor and substrate or product molecules, have been published by our group.

These and other RedAms from fungi have been applied in a number of preparative imine reductions and reductive aminations (Mangas-Sanchez *et al.*, 2020 ▸; Ramsden *et al.*, 2019 ▸; González-Martínez *et al.*, 2020 ▸; Zhang *et al.*, 2021 ▸). One of them, *Ad*RedAm from *Ajellomyces dermatitidis*, has been reported to be more stable than *Asp*RedAm (Zachos *et al.*, 2021 ▸) and thus perhaps more suitable for process applications. Indeed, *Ad*RedAm has already been applied to the asymmetric reduction of imine substrates, including dibenzazepines such as **4** (Fig. 1 ▸ *b*; France *et al.*, 2017 ▸) and pyrrolidines such as **6** (Fig. 1 ▸ *c*; Costa *et al.*, 2018 ▸), and also the reductive amination of cyclohexanone (**1**) with various amines (Aleku *et al.*, 2017 ▸). In addition, *Ad*RedAm has been used in a biocatalytic flow system for the reductive amination of hydrocinnamaldehyde (**8**) with allylamine (**9**) (Fig. 1 ▸ *d*; Finnigan *et al.*, 2020 ▸). Given the enduring interest in *Ad*RedAm as a catalyst, we have determined its structure using X-ray crystallography to assist in the interpretation of experimental biotransformation results and also for structure-guided engineering. The structure, which has been obtained in three crystal forms, sheds light on the conformational dynamics of the enzyme, cofactor binding, and also the molecular determinants of stereoselectivity in the transformations of the substrate 2,2-difluoroacetophenone.

## 2. Materials and methods

### 2.1. Macromolecule production

Cloning and expression of the gene encoding *Ad*RedAm and purification of the protein have been reported previously (Aleku *et al.*, 2017 ▸). The protein, which was purified using nickel-affinity chromatography followed by size-exclusion chromatography, was concentrated to 40 mg ml^−1^ using centrifugal concentrators with a molecular-weight cutoff of 10 kDa.

### 2.2. Crystallization

Concentrated protein at 40 mg ml^−1^ was subjected to crystallization trials in a range of commercial screens in 96-well plate format, using 150 nl protein solution and 150 nl precipitant solution and a Mosquito robot (SPT Labtech). Crystals for data set 1 were harvested from conditions consisting of 0.1 *M* PCTP buffer (sodium propionate, sodium cacodylate trihydrate, bis-Tris propane) pH 4.0, 25% PEG 1500 with the protein pre-complexed with 2 m*M* NADPH_4_. Crystals for data set 2 were recovered from conditions consisting of 0.1 *M* MES buffer pH 6.0, 0.2 *M* MgCl_2_, 20% PEG 6000 with the protein pre-complexed with 2 m*M* NADP^+^. Crystals for data set 3 were grown in 100 m*M* phosphate buffer pH 8.6, 0.2 *M* MgCl_2_, 20%(*w*/*v*) PEG 3350 with the protein pre-complexed with 2 m*M* NADP^+^ and 10 m*M* 2,2-difluoroacetophenone. Crystallization information is summarized in Table 1 ▸.

### 2.3. Data collection and processing

Data were collected on beamlines I03 and I04-1 at the Diamond Light Source (DLS) and were processed and integrated using *XDS* (Kabsch, 2010 ▸) and scaled using *SCALA* (Evans, 2006 ▸) within the *xia*2 (Winter, 2010 ▸) processing system. Data-collection statistics can be found in Table 2 ▸.

### 2.4. Structure solution and refinement

Crystals furnishing data set 1 were obtained in space group *P*3_1_21 with two molecules in the asymmetric unit constituting one dimer. The structure was solved with *MOLREP* (Vagin & Teplyakov, 2010 ▸) using the structure of the imine reductase from *A. oryzae* (54% sequence identity; PDB entry 5g6r; Aleku *et al.*, 2017 ▸) as the molecular-replacement model. The structure was solved using iterative cycles of *Coot* (Emsley *et al.*, 2010 ▸) and *REFMAC* (Murshudov *et al.*, 2011 ▸). After building the protein backbone, side chains and water molecules, residual density was present in the omit map in one active site. This could be modelled and refined as the inactive cofactor molecule NADP_4_. Data sets 2 and 3, in space groups *C*2_1_ and *P*3_1_21, respectively, were processed, built and refined in a similar fashion to data set 1, yielding structures with nine and one monomers in the asymmetric unit, respectively. Data set 2 featured density consistent with nine molecules of NADP^+^ in nine active sites; data set 3 featured density consistent with one molecule of NADP^+^ in its active site, and also residual density adjacent to the nicotinamide ring of the cofactor that was successfully modelled and refined as 2,2-difluoroaceto­phenone (**15**). The Ramachandran plot for the structure from data set 1 revealed 99.6% of residues in favoured regions, with 0.4% outliers. The corresponding figures for data sets 2 and 3 were also 99.6% of residues in favoured regions with 0.4% outliers. Refinement statistics for the structures can be found in Table 3 ▸. Coordinates and structure-factor files have been deposited in the Protein Data Bank (PDB) for data sets 1, 2 and 3 with accession codes 8ozw, 8p2j and 8ozv, respectively.

## 3. Results and discussion

The gene encoding *Ad*RedAm was codon-optimized for expression in *Escherichia coli* and was expressed in *E. coli* BL21 (DE3) cells. The enzyme was purified by nickel-affinity (Ni–NTA) chromatography and size-exclusion chromatography (SEC) using previously described methods (Aleku *et al.*, 2017 ▸), and the pure protein was concentrated to 40 mg ml^−1^ for crystallization. The enzyme crystallized in three forms. The first form (data set 1), which belonged to space group *P*3_1_21 and was refined to 2.01 Å resolution, contained two molecules in the asymmetric unit representing one dimer. The second form (data set 2), which belonged to space group *C*2_1_ and was refined to 1.73 Å resolution, had nine molecules in the asymmetric unit, representing four and a half dimers. The third form (data set 3) belonged to space group *P*3_1_21 and was refined to 1.52 Å resoution. This structure featured only one molecule as a half-dimer in the asymmetric unit. Data-collection and refinement statistics can be found in Tables 2 ▸ and 3 ▸.

**Figure 2:**
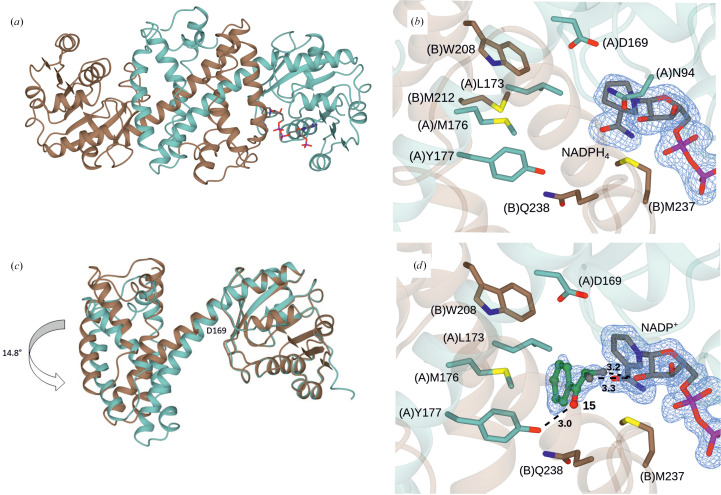
(*a*) Structure of the *Ad*RedAm dimer from data set 1 in ribbon format, with monomers *A* and *B* shown in blue and brown, respectively. (*b*) Active site of *Ad*RedAm showing the active-site residues, each of which is conserved from *Asp*RedAm (PDB entry 5g6r; Aleku *et al.*, 2017 ▸). NADPH_4_ is shown in cylinder format with C atoms in grey. (*c*) Superimposition of monomers *E* and *I* of *Ad*RedAm from data set 2 in blue and brown, respectively. (*d*) Structure of *Ad*RedAm from data set 3. The symmetry neighbour has been incorporated (C atoms in brown) to show the contribution of both monomers in the active site. The ligand 2,2-difluoroacetophenone (**15**) is shown with C atoms in yellow. Electron density in blue corresponds to the unbiased *F*_o_ − *F*_c_ maps at a level of 2.5σ obtained before refinement of the ligand atoms, which have been added for clarity.

Using data set 1, the structure of *Ad*RedAm was solved using the structure of *Asp*RedAm (sequence identity of 54%) as a model. The structure was built and refined using iterative cycles of building in *Coot* and refinement in *REFMAC*. This data set yielded two molecules in the asymmetric unit as a dimer, which is the canonical form of previously described imine reductases (Aleku *et al.*, 2016 ▸, 2017 ▸; Rodríguez-Mata *et al.*, 2013 ▸; Huber *et al.*, 2014 ▸; Man *et al.*, 2015 ▸). Electron density for the backbone atoms was largely complete throughout the length of both chains from Ala2 to Lys288. The monomer of *Ad*RedAm was compared with existing structures using the *DALI* server (Holm, 2022 ▸). Its closest structural homologs were *Asp*RedAm (PDB entry 5g6r; *Z*-score 34.6; 54% sequence identity; r.m.s.d. of 1.0 Å over 289 C^α^ atoms; Aleku *et al.*, 2017 ▸), *Nf*RedAm from *Neosartorya fumigata* (PDB entry 6sle; *Z*-score 33.8; 50% sequence identity; r.m.s.d. of 1.2 Å over 278 C^α^ atoms; Mangas-Sanchez *et al.*, 2020 ▸) and the imine reductase from *Streptosporangium roseum* (PDB entry 5ocm; 39% sequence identity; r.m.s.d. of 1.2 Å over 282 C^α^ atoms; Lenz *et al.*, 2018 ▸). *Ad*RedAm adopts the known IRED fold, with an N-terminal NADP^+^-binding domain (Ala2–Val162) connected by a long inter-domain helix (Gly163–Ser192) to a C-terminal helical bundle (Ala193–Lys288) (Fig. 2 ▸ *a*).

The two *Ad*RedAm monomers associate to form a dimer in which reciprocal domain sharing results in the formation of a large active-site cleft between the N-terminal domain of one monomer and the C-terminal domain of its partner. In the structure from data set 1, following building of the protein and water molecules one of the active sites featured clear density in the omit map that could be modelled as the redox-inactive cofactor NADPH_4_, with which the protein had been complexed (Fig. 2 ▸ *b*). Interestingly, the other active site featured no cofactor density. A comparison of monomers with and without NADPH_4_ showed that the side chain of Asn94 was rotated approximately 180° to accommodate the ribose of the cofactor, but that the orientation of the other side chains was largely conserved.

Despite a sequence homology of only 54%, the active site of *Ad*RedAm is highly conserved compared with that of *Asp*RedAm (for example PDB entry 5g6r; Aleku *et al.*, 2017 ▸), with Asp169 and Asn94, which are thought to have roles in amine activation in the reductive aminase mechanism (Sharma *et al.*, 2018 ▸), and Tyr177, which is implicated in ketone activation, superimposing well with the equivalent residues in *Asp*RedAm (Fig. 2 ▸ *b*). In addition, hydrophobic residues in *Asp*RedAm that were shown to form a binding pocket for ketone substrates (Aleku *et al.*, 2017 ▸), including Leu173 and Met176 from the interdomain helix and Trp208 and Met212 from the C-terminal domain of the partner monomer (monomer *B* in Fig. 2 ▸ *b*), are also conserved, with Met237 and Gln238 at the front of the active site.

The sequences and structures are less conserved in other regions. In the N-terminal domain of *Ad*RedAm there are several differences between Ser60 and His110 when compared with *Asp*RedAm, including Lys70 (Asn69 in *Asp*RedAm), which forms an ionic interaction with Glu102 (Lys101) in *Ad*RedAm. There are also differences at Trp84 (Leu83) and Trp106 (Phe105), residues that both project into the hydrophobic core of the N-terminal domain that also includes Leu77 (Leu76) and Ile89 (Ile88), which are both conserved between the enzymes. In addition, the hydroxyl group of Thr103 in *Ad*RedAm, which is replaced by Leu102 in *Asp*RedAm, forms a new hydrogen bond to the backbone carbonyl group of His99 (Gln98).

However, the major difference in tertiary structure between the *Ad*RedAm and *Asp*RedAm monomers is a shorter loop of 11 residues between Leu189 and Gly199 (LVQSANIPAAG) in the C-terminal helical bundle in *Ad*RedAm, which was 14 residues (LIKSGQDTSTTATG) between Leu189 and Gly202 in *Asp*RedAm. This loop, which is at the dimer interface, positions Ala193 and Ile195 in *Ad*RedAm for hydrophobic interactions with Leu164 and Leu167 in the partner monomer. Just downstream of this loop, in the region between Val190 and Thr210, Val190 (Ile190), Ile195 (Asp195), Leu189 (Leu189) and Phe200 (Leu203) also make hydrophobic interactions with Ala171 and Leu172 of the neighbouring monomer at the dimer interface. Recent studies of the engineering of IREDs for improved stability using random mutagenesis suggest that mutations that enhance interactions, including hydrophobic forces, at the dimer interface were significant in producing variants with greater process stability (Schober *et al.*, 2019 ▸; Kumar *et al.*, 2021 ▸; Ma *et al.*, 2021 ▸) and, indeed, previous research has suggested that *Ad*RedAm is more stable than *Asp*RedAm (Zachos *et al.*, 2021 ▸). A comparative analysis of *Asp*RedAm and *Ad*RedAm using *PISA* (Krissinel & Henrick, 2007 ▸) suggests that *Ad*RedAm should be more stable, with a monomer–monomer interfacial interaction of 4107 Å^2^ versus 3918 Å^2^. This would suggest a free energy of dissociation of −84.8 kJ mol^−1^ for *Ad*RedAm versus −78.3 kJ mol^−1^ for *Asp*RedAm and thus greater stability, as observed experimentally.

The *Ad*RedAm dimer observed in the structure from data set 1 was already instructive in showing two possible states of the monomer in which the non-natural cofactor molecule was either absent or bound. Variations were also readily observed amongst the four dimers present in the structure from data set 2, which was obtained from crystals that grew in space group *C*2_1_ and featured nine molecules in the asymmetric unit. In this structure, once again, the vast majority of backbone atoms could be modelled in subunits *A*–*I* from, in some cases, the leucine and phenylalanine residues in the purification tag at positions −5 and −4 through to Lys288. The exception was chain *H*, in which electron density for Gly229–Gly232 was poor and could not be modelled. In the case of data set 2, all monomers featured electron density in the omit maps that could be modelled as the cofactor NADP^+^. Despite the presence of the cofactor in all monomers, the difference in the conformation of some monomers was pronounced. In the most divergent examples, monomers *E* and *I* exhibited a hinge movement between the N-terminal and C-terminal domains of 14.8° as calculated using the *DynDom* server (Fig. 2 ▸ *c*; Lee *et al.*, 2003 ▸), with the hinge movement centred around the pendant aspartate residue Asp169. This was comparable to the most pronounced difference in conformation observed in multiple dimer structures of *At*RedAm (Sharma *et al.*, 2018 ▸). The overall effect of the hinge movement is to close the active site with respect to the cofactor, presumably to provide the hydrophobic environment that is required to favour greater stability of the transient imine intermediate.

**Figure 3:**
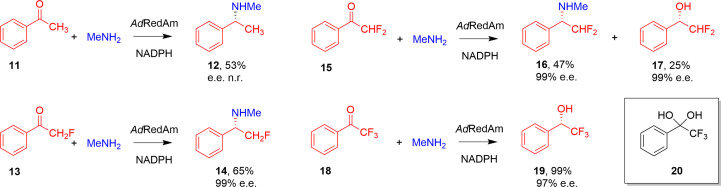
Biotransformation of acetophenone (**11**) and the fluorinated derivatives **13**, **15** and **18** by *Ad*RedAm in the presence of NADPH and methylamine (MeNH_2_) (n.r., not recorded; González-Martínez *et al.*, 2020 ▸). Also shown is the hydrate **20** of **18** that is observed in the active site of the IRED from *S. roseum* (PDB entry 5ocm; Lenz *et al.*, 2018 ▸).

The structure that resulted from data set 3 was more unusual, although not unique amongst IRED structures, in featuring only one monomer (for example PDB entry 6skx; Mangas-Sanchez *et al.*, 2020 ▸) or a half-dimer within the asymmetric unit. However, this data set provides significant further information on ligand recognition within *Ad*RedAm. This structure was obtained from crystals that had been co-crystallized with the ligand 2,2-difluoroacetophenone (**15**; Fig. 3 ▸) in an effort to shed light on the mixed selectivity of *Ad*RedAm towards fluorinated acetophenones, for which both alcohol products and reductive amination products are observed (González-Martínez *et al.*, 2020 ▸). Fluorinated acetophenones are unusual amongst IRED substrates as, with the exception of some examples using engineered enzymes (Jia *et al.*, 2021 ▸), these ketones are the only examples of carbonyl compounds that undergo significant reduction to the alcohol (González-Martínez *et al.*, 2020 ▸; Lenz *et al.*, 2017 ▸).

Indeed, when presented with **15**, the reduced cofactor NADPH and methylamine, *Ad*RedAm catalyzes the formation of the (*S*)-alcohol product **16** and the (*S*)-amine product **17** in 47% and 25% yield, respectively (Fig. 3 ▸), whereas the reduction of acetophenone (**11**) or 2,2,2-trifluoroacetophenone (**18**) under the same conditions gave only the *N*-methyl (**12**) or alcohol products (**19**), respectively (González-Martínez *et al.*, 2020 ▸).

A previous structure of an IRED from *S. roseum* reported by our group (*Sr*IRED; PDB entry 5ocm Lenz *et al.*, 2018 ▸) was presented in complex with the hydrate **20** of ketone **18** and suggested that the significant disposition towards ketone reduction in the case of **18** may be due not only to the extra activation of the carbonyl C atom, but also to aspects of specific substrate recognition within the active site. The complex showed that the fluorine substituents of **20** made hydrogen-bonding interactions with the O2D hydroxyl of the NADP^+^ ribose, thus drawing the electrophilic C atom of the substrate C=O group sufficiently close to the C4 atom of the cofactor for hydride exchange to occur.

For the structure from data set 3, once the protein, water and cofactor atoms had been modelled, significant omit density persisted within the active site that could be modelled as the added ketone ligand 2,2-difluoroacetophenone (**15**; Fig. 2 ▸ *d*) and not as the hydrate form observed for *Sr*IRED with **20**. In the complex of *Ad*RedAm with **15**, the phenyl ring of the substrate is stacked against the side chain of Met176; the carbonyl group of the ketone is coordinated to the phenolic hydroxyl of the side chain of Tyr177 at a distance of 3.0 Å. The F atoms are again positioned 3.2 and 3.3 Å from the O2D atom of the ribose sugar, suggesting again that these inter­actions may be significant in permitting the reduction of the ketone by the cofactor to some extent, but only in the case where F atoms are present. However, the electrophilic C atom is not as ideally placed for hydride exchange as observed for **20** in PDB entry 5ocm, as the distance of the electrophilic C atom from the NADP^+^ atom is suboptimal at 4.5 Å. In addition, the ketone **15** presents its *re* face to the cofactor, which would result in the experimentally observed (*S*)-alcohol product. The complex illustrates the imperfect binding of the fluorinated ketone **15** for either amine or alcohol production, and also provides a basis for understanding the stereoselectivity of *Ad*RedAm for transformations of substrates in this series.

*Ad*RedAm is a useful biocatalyst for a number of imine-reduction and reductive amination reactions. The presented structures of *Ad*RedAm provide new insights into conformational dynamics in this IRED, interactions that confer superior stability and also the basis for steroselectivity and chemo­selectivity in the transformation of fluorinated ketones. In addition to these insights, these and other structures of IREDs will serve as valuable platforms for the structure-guided engineering of IREDs for improved process stability.

## Supplementary Material

PDB reference: imine reductase, data set 1, 8ozw

PDB reference: data set 2, 8p2j

PDB reference: data set 3, 8ozv

## Figures and Tables

**Table 1:** Crystallization

	Data set 1	Data set 2	Data set 3
Method	Sitting drop	Sitting drop	Sitting drop
Plate type	96-well plate	96-well plate	96-well plate
Temperature (K)	298	298	298
Protein concentration (mg ml^−1^)	40	40	40
Buffer composition of protein solution	50 m*M* Tris–HCl buffer pH 7.5, 300 m*M* NaCl	50 m*M* Tris–HCl buffer pH 7.5, 300 m*M* NaCl	50 m*M* Tris–HCl buffer pH 7.5, 300 m*M* NaCl
Composition of reservoir solution	0.1 *M* PCTP pH 4.0, 25% PEG 1500	0.1 *M* MES buffer pH 6.0, 0.2 *M* MgCl_2_, 20% PEG 6000	0.1 *M* phosphate buffer pH 8.6, 0.2 *M* MgCl_2_, 20% PEG 3350
Volume of drop (nl)	150	150	150
Volume of reservoir (µl)	60	60	60

**Table 2:** Data collection and processing Values in parentheses are for the outer shell.

	Data set 1	Data set 2	Data set 3
Diffraction source	I04-1, DLS	I03, DLS	I04-1, DLS
Wavelength (Å)	0.92819	0.97631	0.97950
Temperature (K)	120	120	120
Space group	*P*3_1_21	*C*2_1_	*P*3_1_21
*a*, *b*, *c* (Å)	89.39, 89.39, 136.88	204.62, 87.97, 162.58	74.00, 74.00, 112.08
α, β, γ (°)	90, 90, 120	90, 108.5, 90	90, 90, 120
Resolution range (Å)	77.41–2.01 (2.06–2.01)	97.16–1.73 (1.77–1.73)	42.19–1.52 (1.55–1.52)
No. of unique reflections	42264 (3099)	2781614 (20244)	55313 (2742)
Completeness (%)	98.9 (100.0)	98.8 (96.6)	100.0 (100.0)
Multiplicity	11.4 (12.1)	6.9 (7.1)	12.0 (11.8)
〈*I*/σ(*I*)〉	19.9 (1.9)	11.2 (1.6)	52.1 (24.0)
*R*_r.i.m._	0.11 (1.54)	0.09 (1.16)	0.03 (0.08)
CC_1/2_	1.00 (0.64)	1.00 (0.51)	1.00 (1.00)
Overall *B* factor from Wilson plot (Å^2^)	31	22	10

**Table 3:** Structure refinement Values in parentheses are for the outer shell.

	Data set 1	Data set 2	Data set 3
Resolution range (Å)	77.41–2.01	97.16–1.73	42.19–1.52
Completeness (%)	98.8 (100.0)	98.8 (96.6)	100.0 (100.0)
No. of reflections, working set	40082	264735	52466
No. of reflections, test set	2004	13236	2623
Final *R*_cryst_ (%)	19.3	16.6	13.1
Final *R*_free_ (%)	24.5	19.3	14.7
R.m.s. deviations			
Bond lengths (Å)	0.009	0.009	0.014
Angles (°)	1.63	1.51	1.920
Average *B* factors (Å^2^)			
Protein	35	30	14
Cofactor	30	32	10
Ligand	—	—	34
Water	44	40	31
Ramachandran plot			
Favoured regions (%)	99.6	99.6	99.6
Outliers (%)	0.4	0.4	0.4
