# Structure of the imine reductase from *Ajellomyces dermatitidis* in three crystal forms. Corrigendum

**DOI:** 10.1107/S2053230X25011136

**Published:** 2026-01-01

**Authors:** Mahima Sharma, Anibal Cuetos, Adam Williams, Daniel González-Martínez, Gideon Grogan

**Affiliations:** a Department of Chemistry University of York Heslington YorkYO10 5DD United Kingdom

**Keywords:** imine reductases, biocatalysis, *Ajellomyces dermatitidis*

## Abstract

The name of one of the authors in the article by Sharma *et al.*[(2023), *Acta Cryst.* F**79**, 224–230] is corrected.

## Abstract 2

The article by Sharma *et al.*[(2023), *Acta Cryst.* F**79**, 224–230] is corrected.

In the article by Sharma *et al.* (2023 ▸) the name of one of the authors was given incorrectly. The correct name is Adam Williams as given here.

## References

[bb1] Sharma, M., Cuetos, A., Willliams, A., González-Martínez, D. & Grogan, G. (2023). *Acta Cryst.* F**79**, 224–230. 10.1107/S2053230X23006672 PMC10478762 37581897

